# Systematic review and meta-analysis of depression, anxiety, and suicidal ideation among Ph.D. students

**DOI:** 10.1038/s41598-021-93687-7

**Published:** 2021-07-13

**Authors:** Emily N. Satinsky, Tomoki Kimura, Mathew V. Kiang, Rediet Abebe, Scott Cunningham, Hedwig Lee, Xiaofei Lin, Cindy H. Liu, Igor Rudan, Srijan Sen, Mark Tomlinson, Miranda Yaver, Alexander C. Tsai

**Affiliations:** 1 Center for Global Health, Massachusetts General Hospital, Boston, MA USA; 2 San Mateo County Behavioral Health and Recovery Services, San Mateo, CA USA; 3 Department of Epidemiology and Population Health, Stanford University, Palo Alto, CA USA; 4 Center for Population Health Sciences, Stanford University School of Medicine, Palo Alto, CA USA; 5 Harvard Society of Fellows, Harvard University, Cambridge, MA USA; 6 Department of Electrical Engineering and Computer Science, University of California Berkeley, Berkeley, CA USA; 7 Department of Economics, Hankamer School of Business, Baylor University, Waco, TX USA; 8 Department of Sociology, Washington University in St. Louis, St. Louis, MO USA; 9 Department of Microbiology, Immunology, and Molecular Genetics, Institute for Quantitative and Computational Biosciences, University of California Los Angeles, Los Angeles, CA USA; 10 Departments of Newborn Medicine and Psychiatry, Brigham and Women’s Hospital, Boston, MA USA; 11 Harvard Medical School, Boston, MA USA; 12 Centre for Global Health, Edinburgh Medical School, Usher Institute, University of Edinburgh, Edinburgh, Scotland, UK; 13 Department of Psychiatry, University of Michigan, Ann Arbor, MI USA; 14 Department of Global Health, Institute for Life Course Health Research, Stellenbosch University, Cape Town, South Africa; 15 School of Nursing and Midwifery, Queens University, Belfast, UK; 16 Fielding School of Public Health, Los Angeles Area Health Services Research Training Program, University of California Los Angeles, Los Angeles, CA USA; 17 Mongan Institute, Massachusetts General Hospital, Boston, MA USA

**Keywords:** Epidemiology, Anxiety, Depression, Health policy, Quality of life

## Abstract

University administrators and mental health clinicians have raised concerns about depression and anxiety among Ph.D. students, yet no study has systematically synthesized the available evidence in this area. After searching the literature for studies reporting on depression, anxiety, and/or suicidal ideation among Ph.D. students, we included 32 articles. Among 16 studies reporting the prevalence of clinically significant symptoms of depression across 23,469 Ph.D. students, the pooled estimate of the proportion of students with depression was 0.24 (95% confidence interval [CI], 0.18–0.31; I^2^ = 98.75%). In a meta-analysis of the nine studies reporting the prevalence of clinically significant symptoms of anxiety across 15,626 students, the estimated proportion of students with anxiety was 0.17 (95% CI, 0.12–0.23; I^2^ = 98.05%). We conclude that depression and anxiety are highly prevalent among Ph.D. students. Data limitations precluded our ability to obtain a pooled estimate of suicidal ideation prevalence. Programs that systematically monitor and promote the mental health of Ph.D. students are urgently needed.

## Introduction

Mental health problems among graduate students in doctoral degree programs have received increasing attention^1–4^. Ph.D. students (and students completing equivalent degrees, such as the Sc.D.) face training periods of unpredictable duration, financial insecurity and food insecurity, competitive markets for tenure-track positions, and unsparing publishing and funding models^5–12^—all of which may have greater adverse impacts on students from marginalized and underrepresented populations^13–15^. Ph.D. students’ mental health problems may negatively affect their physical health^16^, interpersonal relationships^17^, academic output, and work performance^18,19^, and may also contribute to program attrition^20–22^. As many as 30 to 50% of Ph.D. students drop out of their programs, depending on the country and discipline^23–27^. Further, while mental health problems among Ph.D. students raise concerns for the wellbeing of the individuals themselves and their personal networks, they also have broader repercussions for their institutions and academia as a whole^22^.

Despite the potential public health significance of this problem, most evidence syntheses on student mental health have focused on undergraduate students^28,29^ or graduate students in professional degree programs (e.g., medical students)^30^. In non-systematic summaries, estimates of the prevalence of clinically significant depressive symptoms among Ph.D. students vary considerably^31–33^. Reliable estimates of depression and other mental health problems among Ph.D. students are needed to inform preventive, screening, or treatment efforts. To address this gap in the literature, we conducted a systematic review and meta-analysis to explore patterns of depression, anxiety, and suicidal ideation among Ph.D. students.

## Results

**Figure 1:**
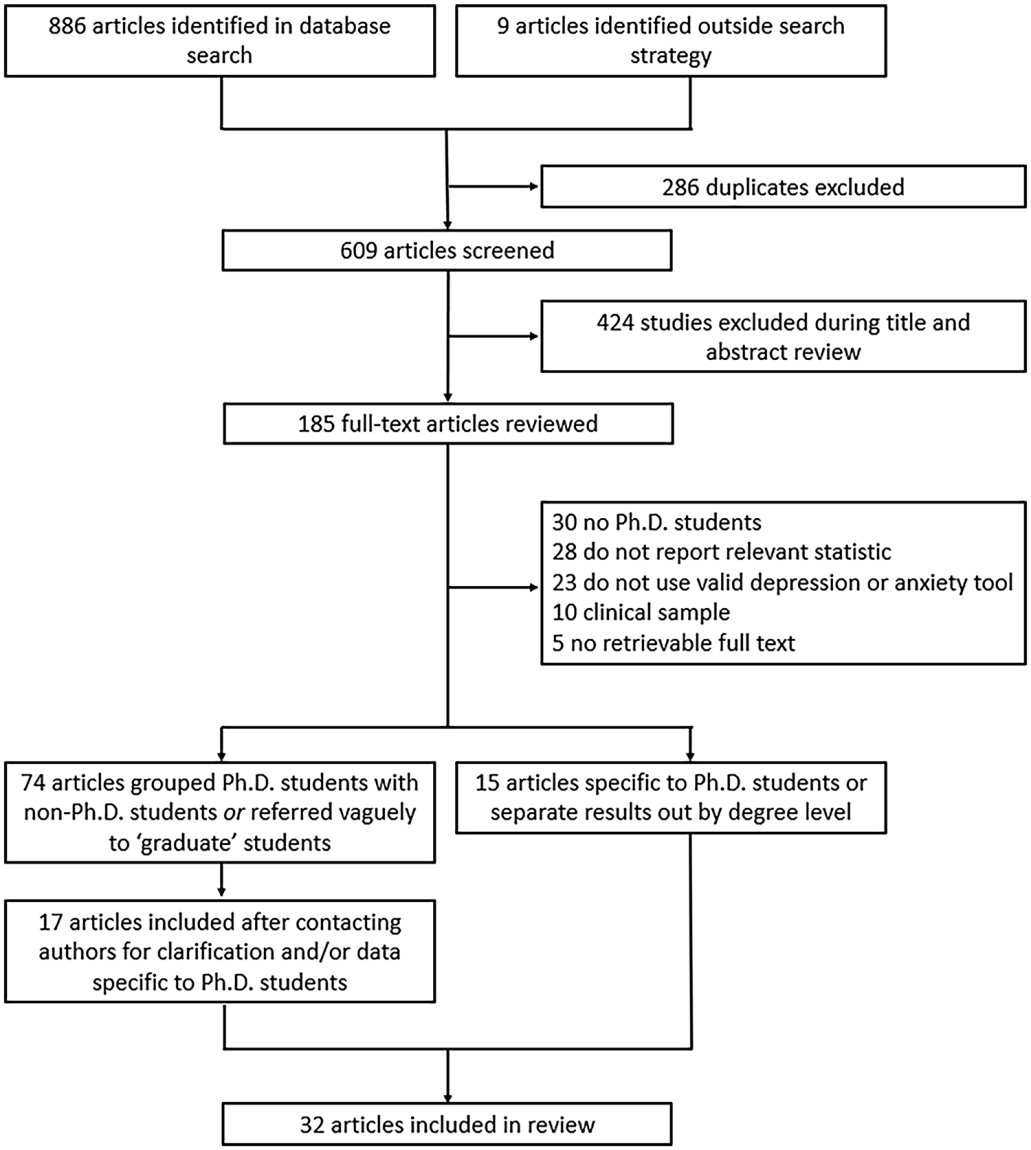
Flowchart of included articles.

The evidence search yielded 886 articles, of which 286 were excluded as duplicates (Fig. 1). An additional nine articles were identified through reference lists or grey literature reports published on university websites. Following a title/abstract review and subsequent full-text review, 520 additional articles were excluded.

Of the 89 remaining articles, 74 were unclear about their definition of graduate students or grouped Ph.D. and non-Ph.D. students without disaggregating the estimates by degree level. We obtained contact information for the authors of most of these articles (69 [93%]), requesting additional data. Three authors clarified that their study samples only included Ph.D. students^34–36^. Fourteen authors confirmed that their study samples included both Ph.D. and non-Ph.D. students but provided us with data on the subsample of Ph.D. students^37–50^. Where authors clarified that the sample was limited to graduate students in non-doctoral degree programs, did not provide additional data on the subsample of Ph.D. students, or did not reply to our information requests, we excluded the studies due to insufficient information (Supplementary Table S1).

Ultimately, 32 articles describing the findings of 29 unique studies were identified and included in the review^16,32–48,50–62^ (Table 1). Overall, 26 studies measured depression, 19 studies measured anxiety, and six studies measured suicidal ideation. Three pairs of articles reported data on the same sample of Ph.D. students^33,38,45,51,53,56^ and were therefore grouped in Table 1 and reported as three studies. Publication dates ranged from 1979 to 2019, but most articles (22/32 [69%]) were published after 2015. Most studies were conducted in the United States (20/29 [69%]), with additional studies conducted in Australia, Belgium, China, Iran, Mexico, and South Korea. Two studies were conducted in cross-national settings representing 48 additional countries. None were conducted in sub-Saharan Africa or South America. Most studies included students completing their degrees in a mix of disciplines (17/29 [59%]), while 12 studies were limited to students in a specific field (e.g., biomedicine, education). The median sample size was 172 students (interquartile range [IQR], 68–654; range, 6–6405). Seven studies focused on mental health outcomes in demographic subgroups, including ethnic or racialized minority students^37,41,43^, international students^47,50^, and sexual and gender minority students^42,54^.

**Table 1:** Summary of included articles.

Author, Year	Country	Sample size (% Female)	Field	Depression			Anxiety			Suicidal ideation		
				Scale (threshold and time frame)	Frequency (prevalence)	Mean (SD)	Scale (threshold and timeframe)	Frequency (prevalence)	Mean (SD)	Scale (timeframe)	Frequency (Prevalence)	Mean (SD)
Bolotnyy, et al. ^52^	USA	513 (34.70%)	Economics	PHQ-9 (≥ 10, past 2 weeks)	91 (17.74%)	NA	GAD-7 (≥ 10, past 2 weeks)	90 (17.60%)	NA	PHQ-9 (past 2 weeks) SBQR (past year)	58 (11.30%) 61 (12.00%)	NA
Baker and Chambers ^44^**	USA	6 (100%)	Social Work	CES-D (≥ 16, past week)	3 (50.00%)	NA	NA	NA	NA	NA	NA	NA
Barry, et al. ^53^ Barry, et al. ^51a^ ^a^ Reported a sample size of 82; adjusted scores are presented	Australia	81 (81.48%) 82 (81.71%)	Mixed	DASS-42 (past week)	NA	6.70 (6.00) 6.71 (5.98)	DASS-42 (past week)	NA	5.80 (6.90) 5.82 (6.92)	NA	NA	NA
Boyle and McKinzie ^54^	USA	632 (NA)	Mixed	DASS-28 (past week)	NA	5.97 (6.86)	DASS-28 (past week)	NA	4.34 (5.65)	NA	NA	NA
Clark, et al. ^37^**	USA	172 (NA)	School Psychology	BSI (past week)	NA	1.51 (0.60)	BSI (past week)	NA	1.47 (0.68)	NA	NA	NA
Corral-Frias, et al. ^49^**	Mexico	7 (NA)	Mixed	MASQ-SF (past week)	NA	20.00 (8.79)	MASQ-SF (past week)	NA	20.00 (7.77)	NA	NA	NA
Eisenberg, et al. ^38^** Golberstein, et al. ^45a^ ^a^ Smaller overall sample size due to missing data	USA	654 (NA)	Mixed	PHQ-9 (≥ 10, past 2 weeks)	91 (13.91%)	NA	PHQ GAD (past 4 weeks)	25 (3.82%)	NA	NCS-R (past 4 weeks) NCS-R (past 4 weeks – plan)	11 (1.67%) 2 (0.30%)	NA
Farrer, et al. ^39^**	Australia	118 (NA)	Mixed	PHQ-9 (≥ 10, past 2 weeks)	13 (11.02%)	5.71 (4.46)	GAD-7 (≥ 10, past 2 weeks)	10 (8.47%)	3.79 (4.23)	NA	NA	NA
Garcia-Williams, et al. ^34^*	USA	301 (77.08%)	Mixed	PHQ-9 (≥ 10, past 2 weeks)	101 (33.55%)	7.95 (5.16)	NA	NA	NA	3-item measure (past 2 weeks)	22 (7.31%)	NA
										3-item measure (lifetime—attempt)	30 (9.97%)	
Heinrich ^55^	USA	68 (NA)	Education	NA	NA	NA	STAI-trait (present)	NA	33.43 (6.86)	NA	NA	NA
Hindman, et al. ^46^**	USA	6 (100.00%)	Mixed	DASS-21 (past week)	NA	3.00 (2.76)	DASS-21 (past week)	NA	2.67 (2.42)	NA	NA	NA
Hirai, et al. ^47^**	USA	46 (NA)	Mixed	DASS-21 (past week)	NA	0.48 (0.59)	DASS-21 (past week)	NA	0.45 (0.45)	NA	NA	NA
Hish, et al. ^56^ Nagy, et al. ^33^	USA	69 (60.87%)	Biomedicine	PHQ-9 (≥ 10, past 2 weeks)	7 (10.14%)	4.64 (4.84)	SCID-5-RV (past year)	22 (31.88%)	NA	NA	NA	NA
Jamshidi, et al. ^57^	Iran	280 (NA)	Medical Sciences	GHQ-28 (past few weeks)	NA	2.56 (4.07)	GHQ-28 (past few weeks)	NA	4.43 (4.05)	NA	NA	NA
Lee and Jeong ^48^**	South Korea	1,809 (NA)	Mixed	BDI (past week)	NA	4.52 (5.15)	NA	NA	NA	NA	NA	NA
Levecque, et al. ^32^	Belgium	3,659 (52.01%)	Mixed	GHQ-12 (≥ 4, past few weeks)	1,165 (31.84%)	NA	NA	NA	NA	NA	NA	NA
Lightstone, et al. ^36^*	USA	116 (77.59%)	Medicine and Psychology	NA	NA	NA	STAI-trait (present)	NA	38.19 (7.87)	NA	NA	NA
Lilly, et al. ^41^**	USA	42 (NA)	Biomedicine	PHQ-2 (past 2 weeks)	3 (7.14%)	NA	NA	NA	NA	NA	NA	NA
Lipson, et al. ^40^** Lipson, et al. ^43^** Lipson, et al. ^42^**^a^ ^a^Due to some overlap in survey years across studies, data pooled (2007–2017)	USA	Total: 13,912 (55.44%)	Mixed	PHQ-9 (≥ 10, past 2 weeks)	Total: 2377/13,912 (17.09%)	Total: 5.83 (4.89)	2007–2012: PHQ GAD (past 4 weeks) 2013–2017: GAD-7 (≥ 10, past 2 weeks)	2007–2012: 326/4,111 (7.93%) 2013–2017: 1,633/9,717 (16.81%)	2007–2012: NA 2013–2017: 5.12 (4.72)	Single-item (past year)	Total: 759/13,660 (5.56%)	NA
Liu, et al. ^58^	China	325 (60.31%)	Medicine	PHQ-9 (≥ 10, past 2 weeks)	77 (23.70%)	NA	GAD-7 (≥ 10, past 2 weeks)	65 (20.00%)	NA	NA	NA	NA
Meghani and Harvey ^50^	USA	32 (NA)	Mixed	Boston X 4 CES-D (≥ 10, past week)	7 (21.88%)	6.43 (5.66)	NA	NA	NA	NA	NA	NA
Richardson, et al. ^62^	USA	119 (85.71%)	Clinical and Counseling Psychology	IDAS-II (past 2 weeks)	NA	46.09 (14.23)	NA	NA	NA	NA	NA	NA
Rummell ^16^	Canada & USA	119 (77.31%)	Clinical and Counseling Psychology	35-item scale derived from DSM-5 (past 2 weeks)	47 (39.29%)	NA	DSM-5 (past 2 weeks)	58 (49.11%)	NA	NA	NA	NA
Sheldon ^35^*	USA	35 (42.86%)	Physics and Ecology	NA	NA	NA	BSI (past week)	NA	0.67 (NA)	NA	NA	NA
Sverdlik and Hall ^59^	54 Countries	3,004 (79.36%)	Mixed	CES-D-10 (past week)	NA	23.31 (4.53)	NA	NA	NA	NA	NA	NA
The Graduate Assembly ^61^	USA	529 (NA)	Mixed	CES-D (≥ 16, past week)	249 (47.07%)	NA	NA	NA	NA	NA	NA	NA
University of California Office of the President ^60^	USA	3,190 (NA)	Mixed	CES-D-R (≥ 16, past 2 weeks)	1,215 (38.09%)	NA	NA	NA	NA	NA	NA	NA

Beck Depression Inventory (BDI), Brief Symptom Inventory (BSI), Center for Epidemiologic Studies–Depression (CES-D), Center for Epidemiologic Studies–Depression–Revised (CES-D-R), Depression Anxiety and Stress Subscales (DASS), Diagnostic and Statistical Manual (DSM), Generalized Anxiety Disorder (GAD), General Health Questionnaire (GHQ), Inventory of Depression and Anxiety Symptoms–Second Version (IDAS-II), Mood and Anxiety Symptom Questionnaire–Short Form (MASQ-SF), National Comorbidity Survey Replication, (NCS-R), Patient Health Questionnaire (PHQ), Suicide Behaviors Questionnaire-Revised (SBQR), State-Trait Anxiety Inventory (STAI), standard deviation (SD), Structured Clinical Interview for DSM-5 Axis I Disorders Research Version (SCID-5-RV).

*Author provided clarification—entire sample consisted of doctoral degree students.

**Author provided additional data—doctoral students reflect a subsample of the total number reported in the published article.

**Figure 2:**
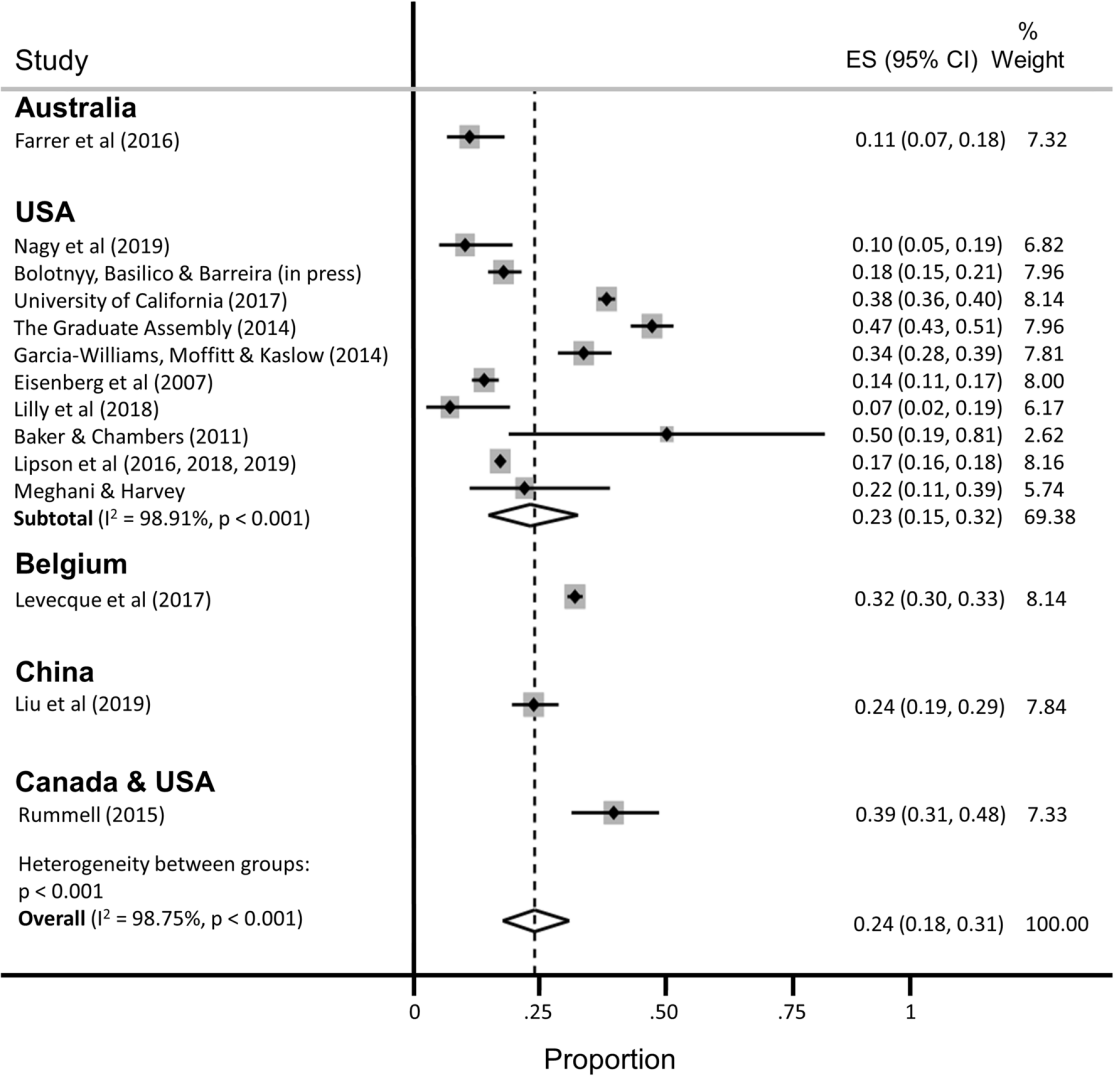
Pooled estimate of the proportion of Ph.D. students with clinically significant symptoms of depression.

In all, 16 studies reported the prevalence of depression among a total of 23,469 Ph.D. students (Fig. 2; range, 10–47%). Of these, the most widely used depression scales were the PHQ-9 (9 studies) and variants of the Center for Epidemiologic Studies-Depression scale (CES-D, 4 studies)^63^, and all studies assessed clinically significant symptoms of depression over the past one to two weeks. Three of these studies reported findings based on data from different survey years of the same parent study (the Healthy Minds Study)^40,42,43^, but due to overlap in the survey years reported across articles, these data were pooled. Most of these studies were based on data collected through online surveys (13/16 [81%]). Ten studies (63%) used random or systematic sampling, four studies (25%) used convenience sampling, and two studies (13%) used multiple sampling techniques.

The estimated proportion of Ph.D. students assessed as having clinically significant symptoms of depression was 0.24 (95% confidence interval [CI], 0.18–0.31; 95% predictive interval [PI], 0.04–0.54), with significant evidence of between-study heterogeneity (I^2^ = 98.75%). A subgroup analysis restricted to the twelve studies conducted in the United States yielded similar findings (pooled estimate [ES] = 0.23; 95% CI, 0.15–0.32; 95% PI, 0.01–0.60), with no appreciable difference in heterogeneity (I^2^ = 98.91%). A subgroup analysis restricted to the studies that used the PHQ-9 to assess depression yielded a slightly lower prevalence estimate and a slight reduction in heterogeneity (ES = 0.18; 95% CI, 0.14–0.22; 95% PI, 0.07–0.34; I^2^ = 90.59%).

**Figure 3:**
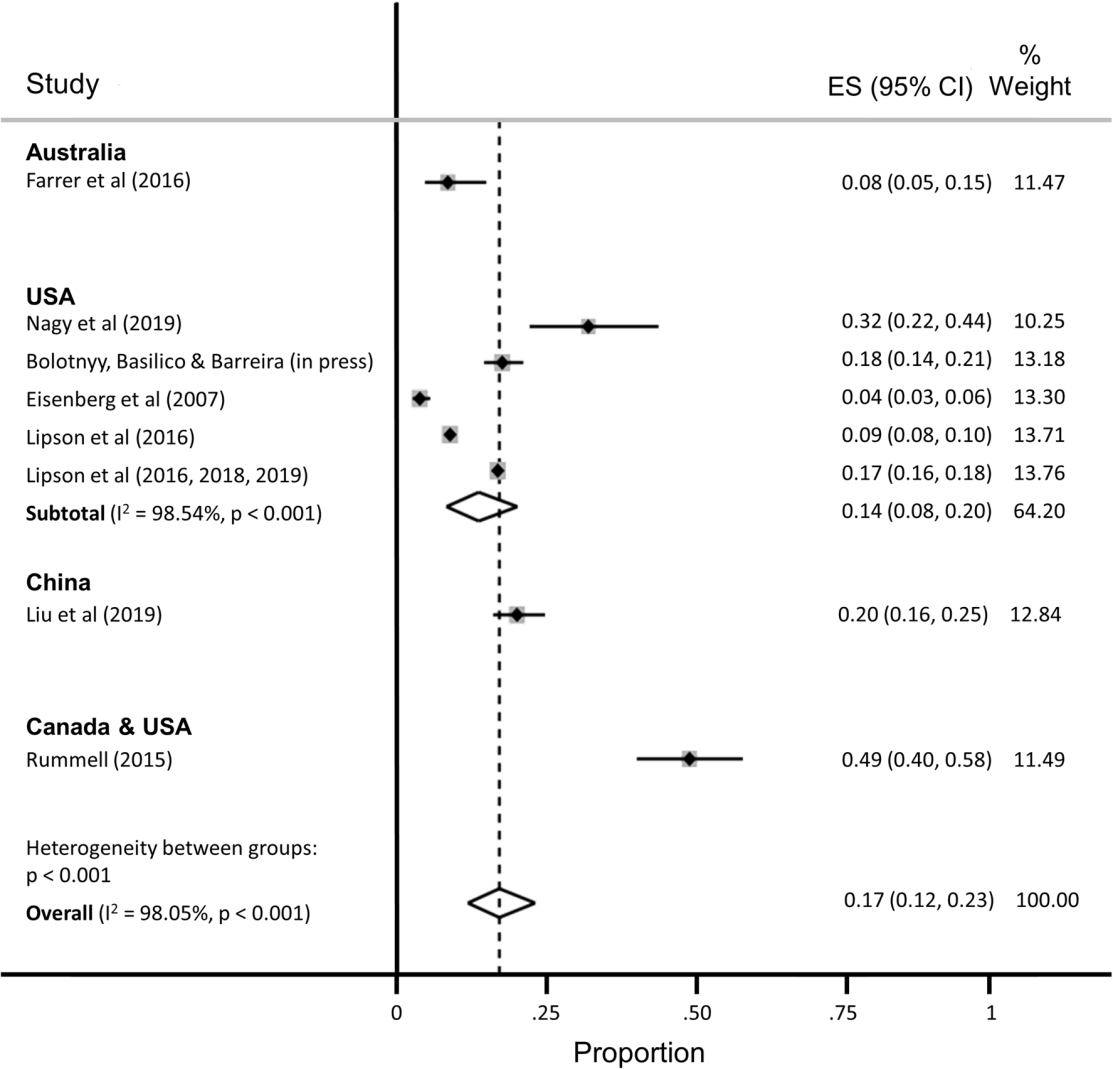
Pooled estimate of the proportion of Ph.D. students with clinically significant symptoms of anxiety.

Nine studies reported the prevalence of clinically significant symptoms of anxiety among a total of 15,626 Ph.D. students (Fig. 3; range 4–49%). Of these, the most widely used anxiety scale was the 7-item Generalized Anxiety Disorder scale (GAD-7, 5 studies)^64^. Data from three of the Healthy Minds Study articles were pooled into two estimates, because the scale used to measure anxiety changed midway through the parent study (i.e., the Patient Health Questionnaire-Generalized Anxiety Disorder [PHQ-GAD] scale was used from 2007 to 2012 and then switched to the GAD-7 in 2013^40^). Most studies (8/9 [89%]) assessed clinically significant symptoms of anxiety over the past two to four weeks, with the one remaining study measuring anxiety over the past year. Again, most of these studies were based on data collected through online surveys (7/9 [78%]). Five studies (56%) used random or systematic sampling, two studies (22%) used convenience sampling, and two studies (22%) used multiple sampling techniques.

The estimated proportion of Ph.D. students assessed as having anxiety was 0.17 (95% CI, 0.12–0.23; 95% PI, 0.02–0.41), with significant evidence of between-study heterogeneity (I^2^ = 98.05%). The subgroup analysis restricted to the five studies conducted in the United States yielded a slightly lower proportion of students assessed as having anxiety (ES = 0.14; 95% CI, 0.08–0.20; 95% PI, 0.00–0.43), with no appreciable difference in heterogeneity (I^2^ = 98.54%).

Six studies reported the prevalence of suicidal ideation (range, 2–12%), but the recall windows varied greatly (e.g., ideation within the past 2 weeks vs. past year), precluding pooled estimation.

Additional stratified pooled estimates could not be obtained. One study of Ph.D. students across 54 countries found that phase of study was a significant moderator of mental health, with students in the comprehensive examination and dissertation phases more likely to experience distress compared with students primarily engaged in coursework^59^. Other studies identified a higher prevalence of mental ill-health among women^54^; lesbian, gay, bisexual, transgender, and queer (LGBTQ) students^42,54,60^; and students with multiple intersecting identities^54^.

Several studies identified correlates of mental health problems including: project- and supervisor-related issues, stress about productivity, and self-doubt^53,62^; uncertain career prospects, poor living conditions, financial stressors, lack of sleep, feeling devalued, social isolation, and advisor relationships^61^; financial challenges^38^; difficulties with work-life balance^58^; and feelings of isolation and loneliness^52^. Despite these challenges, help-seeking appeared to be limited, with only about one-quarter of Ph.D. students reporting mental health problems also reporting that they were receiving treatment^40,52^.

### Risk of bias

Twenty-one of 32 articles were assessed as having low risk of bias (Supplementary Table S2). Five articles received one point for all five categories on the risk of bias assessment (lowest risk of bias), and one article received no points (highest risk). The mean risk of bias score was 3.22 (standard deviation, 1.34; median, 4; IQR, 2–4). Restricting the estimation sample to 12 studies assessed as having low risk of bias, the estimated proportion of Ph.D. students with depression was 0.25 (95% CI, 0.18–0.33; 95% PI, 0.04–0.57; I^2^ = 99.11%), nearly identical to the primary estimate, with no reduction in heterogeneity. The estimated proportion of Ph.D. students with anxiety, among the 7 studies assessed as having low risk of bias, was 0.12 (95% CI, 0.07–0.17; 95% PI, 0.01–0.34; I^2^ = 98.17%), again with no appreciable reduction in heterogeneity.

## Discussion

In our meta-analysis of 16 studies representing 23,469 Ph.D. students, we estimated that the pooled prevalence of clinically significant symptoms of depression was 24%. This estimate is consistent with estimated prevalence rates in other high-stress biomedical trainee populations, including medical students (27%)^30^, resident physicians (29%)^65^, and postdoctoral research fellows (29%)^66^. In the sample of nine studies representing 15,626 Ph.D. students, we estimated that the pooled prevalence of clinically significant symptoms of anxiety was 17%. While validated screening instruments tend to over-identify cases of depression (relative to structured clinical interviews) by approximately a factor of two^67,68^, our findings nonetheless point to a major public health problem among Ph.D. students. Available data suggest that the prevalence of depressive and anxiety disorders in the general population ranges from 5 to 7% worldwide^69,70^. In contrast, prevalence estimates of major depressive disorder among young adults have ranged from 13% (for young adults between the ages of 18 and 29 years in the 2012–2013 National Epidemiologic Survey on Alcohol and Related Conditions III^71^) to 15% (for young adults between the ages of 18 and 25 in the 2019 U.S. National Survey on Drug Use and Health^72^). Likewise, the prevalence of generalized anxiety disorder was estimated at 4% among young adults between the ages of 18 and 29 in the 2001–03 U.S. National Comorbidity Survey Replication^73^. Thus, even accounting for potential upward bias inherent in these studies’ use of screening instruments, our estimates suggest that the rates of recent clinically significant symptoms of depression and anxiety are greater among Ph.D. students compared with young adults in the general population.

Further underscoring the importance of this public health issue, Ph.D. students face unique stressors and uncertainties that may put them at increased risk for mental health and substance use problems. Students grapple with competing responsibilities, including coursework, teaching, and research, while also managing interpersonal relationships, social isolation, caregiving, and financial insecurity^3,10^. Increasing enrollment in doctoral degree programs has not been matched with a commensurate increase in tenure-track academic job opportunities, intensifying competition and pressure to find employment post-graduation^5^. Advisor-student power relations rarely offer options for recourse if and when such relationships become strained, particularly in the setting of sexual harassment, unwanted sexual attention, sexual coercion, and rape^74–78^. All of these stressors may be magnified—and compounded by stressors unrelated to graduate school—for subgroups of students who are underrepresented in doctoral degree programs and among whom mental health problems are either more prevalent and/or undertreated compared with the general population, including Black, indigenous, and other people of color^13,79,80^; women^81,82^; first-generation students^14,15^; people who identify as LGBTQ^83–85^; people with disabilities; and people with multiple intersecting identities.

Structural- and individual-level interventions will be needed to reduce the burden of mental ill-health among Ph.D. students worldwide^31,86^. Despite the high prevalence of mental health and substance use problems^87^, Ph.D. students demonstrate low rates of help-seeking^40,52,88^. Common barriers to help-seeking include fears of harming one’s academic career, financial insecurity, lack of time, and lack of awareness^89–91^, as well as health care systems-related barriers, including insufficient numbers of culturally competent counseling staff, limited access to psychological services beyond time-limited psychotherapies, and lack of programs that address the specific needs either of Ph.D. students in general^92^ or of Ph.D. students belonging to marginalized groups^93,94^. Structural interventions focused solely on enhancing student resilience might include programs aimed at reducing stigma, fostering social cohesion, and reducing social isolation, while changing norms around help-seeking behavior^95,96^. However, structural interventions focused on changing stressogenic aspects of the graduate student environment itself are also needed^97^, beyond any enhancements to Ph.D. student resilience, including: undercutting power differentials between graduate students and individual faculty advisors, e.g., by diffusing power among multiple faculty advisors; eliminating racist, sexist, and other discriminatory behaviors by faculty advisors^74,75,98^; valuing mentorship and other aspects of “invisible work” that are often disproportionately borne by women faculty and faculty of color^99,100^; and training faculty members to emphasize the dignity of, and adequately prepare Ph.D. students for, non-academic careers^101,102^.

Our findings should be interpreted with several limitations in mind. First, the pooled estimates are characterized by a high degree of heterogeneity, similar to meta-analyses of depression prevalence in other populations^30,65,103–105^. Second, we were only able to aggregate depression prevalence across 16 studies and anxiety prevalence across nine studies (the majority of which were conducted in the U.S.) – far fewer than the 183 studies included in a meta-analysis of depression prevalence among medical students^30^ and the 54 studies included in a meta-analysis of resident physicians^65^. These differences underscore the need for more rigorous study in this critical area. Many articles were either excluded from the review or from the meta-analyses for not meeting inclusion criteria or not reporting relevant statistics. Future research in this area should ensure the systematic collection of high-quality, clinically relevant data from a comprehensive set of institutions, across disciplines and countries, and disaggregated by graduate student type. As part of conducting research and addressing student mental health and wellbeing, university deans, provosts, and chancellors should partner with national survey and program institutions (e.g., Graduate Student Experience in the Research University [gradSERU]^106^, the American College Health Association National College Health Assessment [ACHA-NCHA], and HealthyMinds). Furthermore, federal agencies that oversee health and higher education should provide resources for these efforts, and accreditation agencies should require monitoring of mental health and programmatic responses to stressors among Ph.D. students.

Third, heterogeneity in reporting precluded a meta-analysis of the suicidality outcomes among the few studies that reported such data. While reducing the burden of mental health problems among graduate students is an important public health aim in itself, more research into understanding non-suicidal self-injurious behavior, suicide attempts, and completed suicide among Ph.D. students is warranted. Fourth, it is possible that the grey literature reports included in our meta-analysis are more likely to be undertaken at research-intensive institutions^52,60,61^. However, the direction of bias is unpredictable: mental health problems among Ph.D. students in research-intensive environments may be more prevalent due to detection bias, but such institutions may also have more resources devoted to preventive, screening, or treatment efforts^92^. Fifth, inclusion in this meta-analysis and systematic review was limited to those based on community samples. Inclusion of clinic-based samples, or of studies conducted before or after specific milestones (e.g., the qualifying examination or dissertation prospectus defense), likely would have yielded even higher pooled prevalence estimates of mental health problems. And finally, few studies provided disaggregated data according to sociodemographic factors, stage of training (e.g., first year, pre-prospectus defense, all-but-dissertation), or discipline of study. These factors might be investigated further for differences in mental health outcomes.

Clinically significant symptoms of depression and anxiety are pervasive among graduate students in doctoral degree programs, but these are understudied relative to other trainee populations. Structural and clinical interventions to systematically monitor and promote the mental health and wellbeing of Ph.D. students are urgently needed.

## Methods

This systematic review and meta-analysis follows the Preferred Reporting Items for Systematic Reviews and Meta-Analyses (PRISMA) approach (Supplementary Table S3)^107^. This study was based on data collected from publicly available bibliometric databases and did not require ethical approval from our institutional review boards.

### Eligibility criteria

Studies were included if they provided data on either: (a) the number or proportion of Ph.D. students with clinically significant symptoms of depression or anxiety, ascertained using a validated scale; or (b) the mean depression or anxiety symptom severity score and its standard deviation among Ph.D. students. Suicidal ideation was examined as a secondary outcome.

We excluded studies that focused on graduate students in non-doctoral degree programs (e.g., Master of Public Health) or professional degree programs (e.g., Doctor of Medicine, Juris Doctor) because more is known about mental health problems in these populations^30,108–110^ and because Ph.D. students face unique uncertainties. To minimize the potential for upward bias in our pooled prevalence estimates, we excluded studies that recruited students from campus counseling centers or other clinic-based settings. Studies that measured affective states, or state anxiety, before or after specific events (e.g., terrorist attacks, qualifying examinations) were also excluded.

If articles described the study sample in general terms (i.e., without clarifying the degree level of the participants), we contacted the authors by email for clarification. Similarly, if articles pooled results across graduate students in doctoral and non-doctoral degree programs (e.g., reporting a single estimate for a mixed sample of graduate students), we contacted the authors by email to request disaggregated data on the subsample of Ph.D. students. If authors did not reply after two contact attempts spaced over 2 months, or were unable to provide these data, we excluded these studies from further consideration.

### Search strategy and data extraction

PubMed, Embase, PsycINFO, ERIC, and Business Source Complete were searched from inception of each database to November 5, 2019. The search strategy included terms related to mental health symptoms (e.g., depression, anxiety, suicide), the study population (e.g., graduate, doctoral), and measurement category (e.g., depression, Columbia-Suicide Severity Rating Scale) (Supplementary Table S4). In addition, we searched the reference lists and the grey literature.

After duplicates were removed, we screened the remaining titles and abstracts, followed by a full-text review. We excluded articles following the eligibility criteria listed above (i.e., those that were not focused on Ph.D. students; those that did not assess depression and/or anxiety using a validated screening tool; those that did not report relevant statistics of depression and/or anxiety; and those that recruited students from clinic-based settings). Reasons for exclusion were tracked at each stage. Following selection of included articles, two members of the research team extracted data and conducted risk of bias assessments. Discrepancies were discussed with a third member of the research team. Key extraction variables included: study design, geographic region, sample size, response rate, demographic characteristics of the sample, screening instrument(s) used for assessment, mean depression or anxiety symptom severity score (and its standard deviation), and the number (or proportion) of students experiencing clinically significant symptoms of depression or anxiety.

### Risk of bias assessment

Following prior work^30,65^, the Newcastle–Ottawa Scale^111^ was adapted and used to assess risk of bias in the included studies. Each study was assessed across 5 categories: sample representativeness, sample size, non-respondents, ascertainment of outcomes, and quality of descriptive statistics reporting (Supplementary Information S5). Studies were judged as having either low risk of bias (≥ 3 points) or high risk of bias (< 3 points).

### Analysis and synthesis

Before pooling the estimated prevalence rates across studies, we first transformed the proportions using a variance-stabilizing double arcsine transformation^112^. We then computed pooled estimates of prevalence using a random effects model^113^. Study specific confidence intervals were estimated using the score method^114,115^. We estimated between-study heterogeneity using the I^2^ statistic^116^. In an attempt to reduce the extent of heterogeneity, we re-estimated pooled prevalence restricting the analysis to studies conducted in the United States and to studies in which depression assessment was based on the 9-item Patient Health Questionnaire (PHQ-9)^117^. All analyses were conducted using Stata (version 16; StataCorp LP, College Station, Tex.). Where heterogeneity limited our ability to summarize the findings using meta-analysis, we synthesized the data using narrative review.

## Supplementary Information

Supplementary Information.
