# Supplementary material for: Systematic review and meta-analysis of depression, anxiety, and suicidal ideation among Ph.D. students
Source: Sci Rep. 2021 Jul 13;11:14370. doi: 10.1038/s41598-021-93687-7 (PMC8277873; doi:10.1038/s41598-021-93687-7)
Supplement: Supplementary file 1 — Supplementary Information. [file 41598_2021_93687_MOESM1_ESM.pdf]

Supplementary Information

Supplementary Table S1: Articles excluded after contacting authors for additional information

Reason for Exclusion	Author, Year
Authors not contacted: could not obtain valid e-mail address	Brett, Brett, Shaw[118]<br>Byerly, Carlson[119]<br>Dao, Lee, Chang[120]<br>Dillbeck[121]<br>Pallos, Yamada, Okawa[122]
Authors contacted: no reply after two contact attempts	Bahrami, Rajaeepour, Ashrafi Rizi, Zahmatkesh, Nematolahi[123]<br>Baoer, Jiubo, Laiquan, Xueling, Xiaoyuan, Wanjun, Jingbo, Jie[124]<br>Barton, Bulmer[125]<br>Chen, Szalacha, Menon[126]<br>Eremsoy, Celimli, Gencoz[127]<br>Faramarzi, Khafri[128]<br>Fernie, Kopar, Fisher, Spada[129]<br>Ghaderi, Venkatesh Kumar, Kumar[130]<br>Ghaderi, Rangaiah[131]<br>Graham, West, Roemer[132]<br>Klein, Ciotoli, Chung[133]<br>Lyoo, Ju, Kim, Kim, Lee[134]<br>Mokhtari, Jamaluddin, Saad[135]<br>Negi, Khanna, Aggarwal[136]<br>Rice, Choi, Zhang, Morero, Anderson[137]<br>Toews, Lockyer, Dobson, Simpson, Brownell, Brenneis, MacPherson, Cohen[138]<br>Torres, Driscoll, Burrow[139]<br>Verdi, Weyandt, Martinez Zavras[140]<br>Yary[141]<br>Yary, Aazami[142]
Authors contacted, reply received: authors clarified they did not collect data on degree level and therefore could not provide disaggregated data	Pendi, Ashraf, Wolitzky-Taylor, El Magd, Gohar, Khalil, Tsai, Liu, Lee, Baron[143]<br>Bernanke, Galfalvy, Mortali, Hoffman, Moutier, Nemeroff, Stanley, Clayton, Harkavy-Friedman, Oquendo[144]<br>Drum, Brownson, Denmark, Smith[145]<br>Isato, Nishimura, Yamada, Mochizuki[146]<br>Joeng, Turner, Kim, Choi, Kim, Lee[147]<br>Mori, Takano, Tanno[148]<br>Nahidi, Blignault, Hayen, Razee[149]<br>Rossi, Mebert[150]
Authors contacted, reply received: authors clarified the sample did not include doctoral degree students	Hoyer, Gloster, Herzberg[151]<br>Islam, Hossin[152]<br>Kausar, Khan, Rasool, Yusuf, Spielberger[153]

	Khushde, Farhangi, Kouteh, Zahrazei, Ziapour[154]<br>Moritz, Schwarzbold, Guarnieri, Diaz, AL, Dafre[155]<br>Pal, Shepherd, Hamid, Hautus[156]<br>Picardi, Caroppo, Toni, Bitetti, Di Maria[157]<br>Picardi, Toni, Caroppo[158]<br>Saint Arnault, Kim[159]<br>Sharma[160]<br>Tement, Pahor, Jausovec[161]<br>Utsey, McCarthy, Eubanks, Adrian[162]<br>Wang, Xiong, Yang[163]<br>Yamasaki, Uchida, Katsuma[164]
Authors contacted, reply received: authors unable to access data	Armstrong Jr., Goldenberg, Stewart[165]<br>Fraenza[166]<br>Helmers, Danoff, Steinert, Leyton, Young[167]<br>Meeten, Dash, Scarlet, Davey[168]<br>Nezu, Nezu[169]<br>Peluso, Carleton, Asmundson[170]<br>Sheaves, Porcheret, Tsanas, Espie, Foster, Freeman, Harrison, Wulff, Goodwin[171]<br>Valle, DeGood[172]
Authors contacted, reply received: authors declined to provide additional information	Evans, Bira, Gastelum, Weiss, Vanderford[173]<br>Stecker[174]

Supplementary Table S2: Risk of Bias assessment

Author, Year	Sample Representativeness	Sample Size	Non-Respondents	Ascertainment of Outcome	Quality of Descriptive Statistics	Total
Bolotnyy, Basilico, Barreira[52]	1	1	0	1	1	4
Baker, Chambers[44]	0	0	0	1	0	1
Barry, Woods, Warnecke, Stirling, Martin[53]	0	0	0	1	1	2
Barry, Woods, Martin, Stirling, Warnecke[51]	0	0	0	1	1	2
Boyle, McKinzie[54]	0	1	1	1	1****	4
Clark, Mercer, Zeigler-Hill, Dufrene[37]	1	1**	0	1	1****	4
Corral-Frías, Velardez-Soto, Frías-Armenta, Corona-Espinosa, Watson[49]	1*	1**	0	1	1****	4
Eisenberg, Gollust, Golberstein, Hefner[38]	0	1	1***	1	1****	4
Farrer, Gulliver, Bennett, Fassnacht, Griffiths[39]	0	1**	1***	1	1****	4
Garcia-Williams, Moffitt, Kaslow[34]	0	1	1	1	1	4
Golberstein, Eisenberg, Gollust[45]	0	1	1***	1	1****	4
Heinrich[55]	0	0	0	1	0	1
Hindman, Glass, Arnkoff, Maron[46]	0	0	0	1	1****	2
Hirai, Frazier, Syed[47]	0	1**	1	1	1****	4
Hish, Nagy, Fang, Kelley, Nicchitta, Dzirasa, Rosenthal[56]	0	0	0	1	1	2

Author, Year	Sample Representativeness	Sample Size	Non-Respondents	Ascertainment of Outcome	Quality of Descriptive Statistics	Total
Nagy, Fang, Hish, Kelly, Nicchitta, Dzirasa, Rosenthal[33]	0	0	0	1	1	2
Jamshidi, Mogehi, Cheraghi, Jafari, Kabi, Rashidi[57]	0	1	1	1	0	3
Lee, Jeong[48]	0	1	0	1	1****	3
Levecque, Anseel, De Beuckelaer, Van der Heyden, Gisle[32]	1	1	1	1	1	5
Lightstone, Swencionis, Cohen[36]	0	0	0	1	1	2
Lilly, Owens, Bailey, Brown, Clawson, Vidal[41]	0	1**	0	0	1****	2
Lipson, Zhou, Wagner III, Beck, Eisenberg[40]	1	1	1***	1	1****	5
Lipson, Kern, Eisenberg, Breland-Noble[43]	1	1	1***	1	1****	5
Lipson, Raifman, Abelson, Reisner[42]	1	1	1***	1	1****	5
Liu, Wang, Qi, Wang, Jia, Shang, Shao, Yu, Zhu, Yan, Chang, Zhao[58]	1	1	0	1	1	4
Meghani, Harvey[50]	1*	0	0	1	1****	3
Richardson, Trusty, George[62]	1	0	0	1	1	3
Rummell[16]	1	0	0	0	1	2
Sheldon[35]	0	0	0	0	0	0
Sverdlik, Hall[59]	1	1	0	1	1	4
The Graduate Assembly[61]	0	1	1***	1	1****	4
University of California	1	1	1***	1	1****	5

Author, Year	Sample Representativeness	Sample Size	Non-Respondents	Ascertainment of Outcome	Quality of Descriptive Statistics	Total
Office of the President[60]						

*Entire sample included students spanning multiple years of study at multiple institutions, but not necessarily among the subset of Ph.D. students
**Entire sample ≥200, but subsample of Ph.D. students <200
***Response rate and/or comparability between respondents and non-respondents reported in publication for entire student sample, but not stratified by degree level
****Descriptive statistics reported for entire student sample, but not stratified by degree level

Supplementary Table S3: Preferred Reporting Items for Systematic Reviews and Meta-Analyses (PRISMA) checklist

Section/topic	#	Checklist item	Reported on page #
**TITLE**			
Title	1	Identify the report as a systematic review, meta-analysis, or both.	1
**ABSTRACT**			
Structured summary	2	Provide a structured summary including, as applicable: background; objectives; data sources; study eligibility criteria, participants, and interventions; study appraisal and synthesis methods; results; limitations; conclusions and implications of key findings; systematic review registration number.	2
**INTRODUCTION**			
Rationale	3	Describe the rationale for the review in the context of what is already known.	3
Objectives	4	Provide an explicit statement of questions being addressed with reference to participants, interventions, comparisons, outcomes, and study design (PICOS).	3
**METHODS**			
Protocol and registration	5	Indicate if a review protocol exists, if and where it can be accessed (e.g., Web address), and, if available, provide registration information including registration number.	NA
Eligibility criteria	6	Specify study characteristics (e.g., PICOS, length of follow-up) and report characteristics (e.g., years considered, language, publication status) used as criteria for eligibility, giving rationale.	16
Information sources	7	Describe all information sources (e.g., databases with dates of coverage, contact with study authors to identify additional studies) in the search and date last searched.	16-17
Search	8	Present full electronic search strategy for at least one database, including any limits used, such that it could be repeated.	Suppl Table S4
Study selection	9	State the process for selecting studies (i.e., screening, eligibility, included in systematic review, and, if applicable, included in the meta-analysis).	16-17
Data collection process	10	Describe method of data extraction from reports (e.g., piloted forms, independently, in duplicate) and any processes for obtaining and confirming data from investigators.	17
Data items	11	List and define all variables for which data were sought (e.g., PICOS, funding sources) and any assumptions and simplifications made.	17
Risk of bias in individual studies	12	Describe methods used for assessing risk of bias of individual studies (including specification of whether this was done at the study or outcome level), and how this information is to be used in any data synthesis.	17-18

Section/topic	#	Checklist item	Reported on page #
Summary measures	13	Summary measures State the principal summary measures (e.g., risk ratio, difference in means).	18
Synthesis of results	14	Describe the methods of handling data and combining results of studies, if done, including measures of consistency (e.g., I2) for each meta-analysis.	16-18
Risk of bias across studies	15	Specify any assessment of risk of bias that may affect the cumulative evidence (e.g., publication bias, selective reporting within studies).	17-18
Additional analyses	16	Describe methods of additional analyses (e.g., sensitivity or subgroup analyses, meta-regression), if done, indicating which were pre-specified.	18
**RESULTS**			
Study selection	17	Give numbers of studies screened, assessed for eligibility, and included in the review, with reasons for exclusions at each stage, ideally with a flow diagram.	4, Figure 1
Study characteristics	18	For each study, present characteristics for which data were extracted (e.g., study size, PICOS, follow-up period) and provide the citations.	4-8
Risk of bias within studies	19	Present data on risk of bias of each study and, if available, any outcome level assessment (see item 12).	11, Suppl Table S2
Results of individual studies	20	For all outcomes considered (benefits or harms), present, for each study: (a) simple summary data for each intervention group (b) effect estimates and confidence intervals, ideally with a forest plot.	4-11, Figure 2, Figure 3
Synthesis of results	21	Present results of each meta-analysis done, including confidence intervals and measures of consistency.	9-4-11
Risk of bias across studies	22	Present results of any assessment of risk of bias across studies (see Item 15).	11, Suppl Table S2
Additional analysis	23	Give results of additional analyses, if done (e.g., sensitivity or subgroup analyses, meta-regression [see Item 16]).	10-11
**DISCUSSION**			
Summary of evidence	24	Summarize the main findings including the strength of evidence for each main outcome; consider their relevance to key groups (e.g., healthcare providers, users, and policy makers).	12-14
Limitations	25	Discuss limitations at study and outcome level (e.g., risk of bias), and at review-level (e.g., incomplete retrieval of identified research, reporting bias).	14-15
Conclusions	26	Provide a general interpretation of the results in the context of other evidence, and implications for future research.	15
**FUNDING**			

Section/topic	#	Checklist item	Reported on page #
Funding	27	Describe sources of funding for the systematic review and other support (e.g., supply of data); role of funders for the systematic review.	19
From: Moher D, Liberati A, Tetzlaff J, Altman DG, The PRISMA Group (2009). Preferred Reporting Items for Systematic Reviews and Meta-Analyses: The PRISMA Statement. PLoS Med 6(7): e1000097. doi:10.1371/journal.pmed1000097			

Supplementary Table S4: Search terms

Mental Health Symptom Category	
Common terms	Depressed<br>Depression<br>Depressive<br>MDD<br>Anxiety<br>Anxious<br>GAD<br>Distress<br>Distressed<br>Burnout<br>Suicide*<br>Suicidal*<br>Mental health
MeSH (PubMed/MEDLINE)	Depression [MeSH]<br>Depressive disorder [MeSH]<br>Depressive disorder, major [MeSH]<br>Anxiety [MeSH]<br>Anxiety disorders [MeSH]<br>Burnout, Psychological [MeSH]<br>Burnout, Professional [MeSH]<br>Suicide [MeSH]<br>Mental Health[MeSH]
Emtree Subject Headings (EMBASE)	Depression<br>Major depression<br>Anxiety<br>Anxiety disorder<br>Generalized anxiety disorder<br>Burnout<br>Professional burnout<br>Student burnout<br>Suicide<br>Suicidal behavior<br>Suicidal ideation<br>Suicide attempt<br>Mental Health

AND

Target Population Category	
Common terms	Graduate<br>Postgraduate<br>Post-graduate<br>PhD<br>Ph.D.<br>Doctoral<br>Predoctoral<br>Pre-doctoral<br>Student*

	Candidate*
MeSH (PubMed/MEDLINE)	
Emtree Subject Headings (EMBASE)	graduate student<br>postgraduate student<br>PhD student<br>research student

AND

Measure Category	
Common terms	Aga Khan University Anxiety and Depression Scale<br>AKUADS<br>Patient Health Questionnaire<br>PHQ*<br>General Health Questionnaire<br>GHQ*<br>Beck<br>BDI*<br>Major Depression Inventory<br>MDI<br>Mood and Anxiety Symptoms Questionnaire<br>MASQ*<br>Montgomery<br>MADRS<br>Hamilton<br>HAM-D<br>HAMD<br>HSRD<br>HDRS<br>Center for Epidemiologic Studies<br>CES-D<br>CESD<br>Depression Anxiety Stress Scale*<br>Depression Anxiety and Stress Scale*<br>DASS*<br>Zung<br>SDS<br>ZSDS<br>Quick Inventory of Depressive Symptomatology<br>QIDS<br>Clinically Useful Depression Outcome Scale<br>CUDOS<br>Generalized Anxiety Disorder<br>GAD*<br>BAI<br>HAM-A<br>HAMA<br>SAS<br>ZSAS<br>Primary Care Evaluation of Mental Disorders<br>PRIME-MD

	PRIMEMD<br>Brief Symptom Inventory<br>BSI<br>Derogatis Stress Profile<br>DSP<br>Hospital Anxiety and Depression Scale<br>HADS<br>Suicide Behavio?rs Questionnaire<br>SBQ*<br>State-Trait Anxiety Inventory<br>STAI<br>Columbia Suicide Severity Rating Scale<br>Columbia suicide screen<br>SSRS<br>CSSRS<br>Structured Clinical Interview<br>SCID<br>Prevalence<br>Incidence<br>(standardized OR validated OR structured OR diagnostic OR screening) AND (questionnaire? OR survey? OR interview? OR instrument? OR measure?)
MeSH (PubMed/MEDLINE)	Patient health questionnaire [MeSH]<br>Psychiatric Rating Scales [MeSH]<br>Prevalence [MeSH]<br>Incidence [MeSH]
Emtree Subject Headings (EMBASE)	Beck Depression Inventory<br>Center for Epidemiological Studies Depression Scale<br>Depression Inventory<br>Hamilton Depression Rating Scale<br>Montgomery Asberg Depression Rating Scale<br>Self-rating Depression Scale<br>Zung Self Rating Depression Scale<br><br>Patient Health Questionnaire<br>General Health Questionnaire<br>Depression Anxiety Stress Scale<br>Hospital Anxiety and Depression Scale<br>Brief Symptom Inventory<br>Structured Clinical Interview for DSM Disorders<br><br>Beck Anxiety Inventory<br>Hamilton Anxiety Scale<br>Self-rating Anxiety Scale<br>State Trait Anxiety Inventory<br>Prevalence<br>Incidence

Supplementary Information S5: Modified Newcastle-Ottawa Risk of Bias scoring guide

We followed the approach of Rotenstein, Ramos, Torre, Segal, Peluso, Guille, Sen, Mata[30] and Mata, Ramos, Bansal, Khan, Guille, De Angelantonio, Sen[65] in adapting the Newcastle-Ottawa Scale[111] to assess risk of bias in the included studies. Studies received one point for 'sample representativeness' if the sample contained Ph.D. students spanning multiple years of study and students attending multiple schools. For 'sample size', studies received one point if they included ≥200 Ph.D. students. Studies received one point for 'non-respondents' if comparability between respondent and non-respondent characteristics was established with a satisfactory response rate. If studies employed a commonly used measurement tool with a validated cutoff score, they received one point for 'ascertainment of outcome'. Finally, if studies reported descriptive statistics to describe the population of Ph.D. students (e.g., age, sex, race) with some measures of dispersion, studies received one point for 'quality of descriptive statistics reporting'.

The individual components were summed to generate a total modified Newcastle-Ottawa Scale risk of bias score, ranging from 0 to 5, for each study. Again, following the score thresholds in Rotenstein, Ramos, Torre, Segal, Peluso, Guille, Sen, Mata[30] and Mata, Ramos, Bansal, Khan, Guille, De Angelantonio, Sen[65], each study was categorized as having either low risk of bias (≥3 points) or high risk of bias (<3 points).

(1) Representativeness of sample*:

1 point: Sample contained students spanning multiple years of study at multiple institutions
0 points: Sample contained students in either a single year of study or at a single institution

(2) Sample size*:

1 point: Sample included ≥200 students
0 points: Sample included <200 students

(3) Non-respondents*:

1 point: Comparability between respondent and non-respondent characteristics was established, or the response rate was 95% or greater
0 points: The comparability between respondents and non-respondents was unsatisfactory, the response rate was unsatisfactory (<95%), or there was no description of the response rate or the characteristics of the responders and non-responders

(4) Ascertainment of outcome:

1 point: Study employed a well described and/or validated measurement tool
0 points: Study did not employ a well described and/or validated measurement tool

(5) Quality of descriptive statistics reporting*:

1 point: Study reported descriptive statistics to describe the population (e.g., age, sex) with proper measures of dispersion (e.g., mean, standard deviation)

0 points: Descriptive statistics were not reported, were incomplete, or did not include proper measures of dispersion

***** Since a subset of articles pooled data between students in doctoral and non-doctoral degree programs, our risk of bias assessments were conditioned on the study aims. For example, some studies included 200 or more graduate students in their pooled sample, but fewer than 200 Ph.D. students specifically. Other studies presented data on the comparability of respondents and non-respondents only for the pooled sample. These studies received one point on the risk of bias assessment.

Supplementary Information References

118. Brett CA, Brett AS, Shaw SS. Impact of traumatic incidents on family-of-origin functioning: An empirical study. *J Contemp Psychother.* 1993;23(4):255-266.
119. Byerly FC, Carlson WA. Comparison among inpatients, outpatients, and normals on three self-report depression inventories. *J Clin Psychol.* 1982;38(4):797-804.
120. Dao TK, Lee D, Chang HL. Acculturation level, perceived English fluency, perceived social support level, and depression among Taiwanese international students. *Coll Stud J.* 2007;41(2):287-295.
121. Dillbeck MC. The effect of the Transcendental Meditation technique on anxiety level. *J Clin Psychol.* 1977;33(4):1076-1078.
122. Pallos H, Yamada N, Okawa M. Graduate student blues. *J College Stud Psychother.* 2005;20(2):5-15.
123. Bahrami S, Rajaeepour S, Ashrafi Rizi H, Zahmatkesh M, Nematolahi Z. The relationship between students' study habits, happiness and depression. *Iran J Nurs Midwifery Res.* 2011;16(3):217-221.
124. Baoer Z, Jiubo Z, Laiquan Z, et al. Depressive symptoms, post-traumatic stress symptoms and suicide risk among graduate students: The mediating influence of emotional regulatory self-efficacy. *Psychiatry Res.* 2018;264:224-230.
125. Barton BA, Bulmer SM. Correlates and predictors of depression and anxiety disorders in graduate students. *Health Educator.* 2017;49(2):17-26.
126. Chen AC, Szalacha LA, Menon U. Perceived discrimination and its associations with mental health and substance use among Asian American and Pacific Islander undergraduate and graduate students. *J Am Coll Health.* 2014;62(6):390-398.
127. Eremsoy CE, Celimli S, Gencoz T. Students under academic stress in a Turkish university: Variables associated with symptoms of depression and anxiety. *Curr Psychol.* 2005;24(2):123-133.
128. Faramarzi M, Khafri S. Role of alexithymia, anxiety, and depression in predicting self-efficacy in academic students. *ScientificWorldJournal.* 2017;2017:5798372.
129. Fernie BA, Kopar UY, Fisher PL, Spada MM. Further development and testing of the metacognitive model of procrastination: Self-reported academic performance. *J Affect Disord.* 2018;240:1-5.
130. Ghaderi A, Venkatesh Kumar G, Kumar S. Depression, anxiety and stress among the Indian and Iranian students. *J Indian Acad Appl Psychol.* 2009;35(1):33-37.
131. Ghaderi A, Rangaiah B. Influence of self-efficacy on depression, anxiety and stress among Indian and Iranian students. *J Psychosoc Res.* 2011;6(2):231-240.
132. Graham JR, West L, Roemer L. A preliminary exploration of the moderating role of valued living in the relationships between racist experiences and anxious and depressive symptoms. *J Context Behav Sci.* 2015;4(1):48-55.
133. Klein MC, Ciotoli C, Chung H. Primary care screening of depression and treatment engagement in a university health center: A retrospective analysis. *J Am Coll Health.* 2011;59(4):289-295.
134. Lyoo YC, Ju S, Kim E, Kim JE, Lee JH. The patient health questionnaire-15 and its abbreviated version as screening tools for depression in Korean college and graduate students. *Compr Psychiatry.* 2014;55(3):743-748.
135. Mokhtari T, Jamaluddin R, Saad HA. Lifestyle and psychological factors associated with body weight status among university students in Malaysia. *Pak J Nutrition.* 2015;14(1):18-28.
136. Negi AS, Khanna A, Aggarwal R. Psychological health, stressors and coping mechanism of engineering students. *Int J Adolesc Youth.* 2019;24(4):511-520.

137. Rice KG, Choi C-C, Zhang Y, Morero YI, Anderson D. Self-critical perfectionism, acculturative stress, and depression among international students. *Counsel Psychol.* 2012;40(4):575-600.
138. Toews JA, Lockyer JM, Dobson DJG, et al. Analysis of stress levels among medical students, residents, and graduate students at four Canadian schools of medicine. *Acad Med.* 1997;72(11):997-1002.
139. Torres L, Driscoll MW, Burrow AL. Racial microaggressions and psychological functioning among highly achieving African-Americans: A mixed-methods approach. *J Soc Clin Psychol.* 2010;29(10):1074-1099.
140. Verdi G, Weyandt LL, Martinez Zavras B. Non-medical prescription stimulant use in graduate students: Relationship with academic self-efficacy and psychological variables. *J Atten Disord.* 2016;20(9):741-753.
141. Yary T. The association between dietary intake of folate and physical activity with psychological dimensions of depressive symptoms among students from Iran. *BioMed Res Int.* 2013;2013(582693):1-10.
142. Yary T, Aazami S. The association between polyunsaturated fatty acids and depression among Iranian postgraduate students in Malaysia. *Lipids Health Dis.* 2011;10(151).
143. Pendi A, Ashraf J, Wolitzky-Taylor K, et al. Depression and depression-specific stigma in American graduate students at a large metropolitan university: Preliminary findings of a cross-sectional study. 29th Annual U.S. Psychiatric & Mental Health Congress; 2016; San Antonio, Texas.
144. Bernanke J, Galfalvy HC, Mortali MG, et al. Suicidal ideation and behavior in institutions of higher learning: A latent class analysis. *J Psychiatr Res.* 2017;95:253-259.
145. Drum DJ, Brownson C, Denmark AB, Smith SE. New data on the nature of suicidal crises in college students: Shifting the paradigm. *Prof Psychol Res Pr.* 2009;40(3):213-222.
146. Isato A, Nishimura H, Yamada M, Mochizuki S. Inhibition of internal attention to positive and negative representations in dysphoria. *Jap Psychol Res.* 2018;60(3):143-155.
147. Joeng JR, Turner SL, Kim EY, Choi SA, Kim JK, Lee YJ. Data for Korean college students anxious and avoidant attachment, self-compassion, anxiety and depression. *Data Brief.* 2017;13:316-319.
148. Mori M, Takano K, Tanno Y. Role of self-focus in the relationship between depressed mood and problem solving. *Motivation Emotion.* 2015;39(5):827-838.
149. Nahidi S, Blignault I, Hayen A, Razee H. Psychological distress in Iranian international students at an Australian university. *J Immigr Minor Health.* 2018;20(3):651-657.
150. Rossi NE, Mebert CJ. Does a quarterlife crisis exist? *J Genet Psychol.* 2011;172(2):141-161.
151. Hoyer J, Gloster AT, Herzberg PY. Is worry different from rumination? Yes, it is more predictive of psychopathology! *Psychosoc Med.* 2009;6:Doc06.
152. Islam MA, Hossin MZ. Prevalence and risk factors of problematic internet use and the associated psychological distress among graduate students of Bangladesh. *Asian J Gambl Issues Public Health.* 2016;6(11).
153. Kausar R, Khan N, Rasool F, Yusuf S, Spielberger CD. Translation and adaptation of State Trait Anxiety Inventory (STAIY) in Urdu. *J Behav Sci.* 2012;22(3):132-146.
154. Khushde S, Farhangi M, Kouteh BR, Zahrazei F, Ziapour A. A comparative study of homesickness, depression, and internet addiction between native and nonnative

students at University of Sistan and Baluchestan, Iran. *Ann Top Med Public Health.* 2018;10:1537-1546.
155. Moritz B, Schwarzbold ML, Guarnieri R, Diaz AP, AL SR, Dafre AL. Effects of ascorbic acid on anxiety state and affect in a non-clinical sample. *Acta Neurobiol Exp (Wars).* 2017;77(4):362-372.
156. Pal PR, Shepherd D, Hamid N, Hautus MJ. The potential for retronasally delivered olfactory stimuli to assess psychiatric conditions. *Curr Psychol J Diverse Perspect Diverse Psychol Issues.* 2019:No Pagination Specified.
157. Picardi A, Caroppo E, Toni A, Bitetti D, Di Maria G. Stability of attachment-related anxiety and avoidance and their relationships with the five-factor model and the psychobiological model of personality. *Psychol Psychother.* 2005;78(Pt 3):327-345.
158. Picardi A, Toni A, Caroppo E. Stability of alexithymia and its relationships with the 'big five' factors, temperament, character, and attachment style. *Psychother Psychosom.* 2005;74(6):371-378.
159. Saint Arnault D, Kim O. Is there an Asian idiom of distress? Somatic symptoms in female Japanese and Korean students. *Arch Psychiatr Nurs.* 2008;22(1):27-38.
160. Sharma S. Sex differences in self-reported anxiousness for different situations and modes of response among university students in India. *Psychologia Int J Psychol Orient.* 1978;21(3):155-160.
161. Tement S, Pahor A, Jausovec N. EEG alpha frequency correlates of burnout and depression: The role of gender. *Biol Psychol.* 2016;114:1-12.
162. Utsey SO, McCarthy E, Eubanks R, Adrian G. White racism and suboptimal psychological functioning among White Americans: Implications for counseling and prejudice prevention. *J Multicult Couns Devel.* 2002;30(2):81-95.
163. Wang P, Xiong Z, Yang H. Relationship of mental health, social support, and coping styles among graduate students: Evidence from Chinese universities. *Iran J Public Health.* 2018;47(5):689-697.
164. Yamasaki K, Uchida K, Katsuma R. An intervention study of the effects of the coping strategy of "finding positive meaning" on positive affect and health. *Int J Psychol.* 2009;44(4):249-256.
165. Armstrong Jr. HE, Goldenberg E, Stewart D. Correlations between Beck Depression scores and physical complaints. *Psychol Rep.* 1980;46:740-742.
166. Fraenza CB. The role of social influence in anxiety and the imposter phenomenon. *Online Learn J.* 2016;20(2):230-243.
167. Helmers KF, Danoff D, Steinert Y, Leyton M, Young SN. Stress and depressed mood in medical students, law students, and graduate students at McGill University. *Acad Med.* 1997;72(8):708-714.
168. Meeten F, Dash SR, Scarlet AL, Davey GC. Investigating the effect of intolerance of uncertainty on catastrophic worrying and mood. *Behav Res Ther.* 2012;50(11):690-698.
169. Nezu AM, Nezu CM. Psychological distress, problem solving, and coping reactions: Sex role differences. *Sex Roles.* 1987;16(3-4):205-214.
170. Peluso DL, Carleton RN, Asmundson GJG. Depression symptoms in Canadian psychology graduate students: Do research productivity, funding, and the academic advisory relationship play a role? *Can J Behav Sci.* 2011;43(2):119-127.
171. Sheaves B, Porcheret K, Tsanas A, et al. Insomnia, nightmares, and chronotype as markers of risk for severe mental illness: Results from a student population. *Sleep.* 2016;39(1):173-181.
172. Valle RS, DeGood DE. Effects of state-trait anxiety on the ability to enhance and suppress EEG alpha. *Psychophysiol.* 1977;14(1):1-7.

173. Evans TM, Bira L, Gastelum JB, Weiss LT, Vanderford NL. Evidence for a mental health crisis in graduate education. *Nat Biotechnol.* 2018;36(3):282-284.
174. Stecker T. Well-being in an academic environment. *Med Educ.* 2004;38:465-478.
